# Supplementary material for: Structural insights into p300 regulation and acetylation-dependent genome organisation
Source: Nat Commun. 2022 Dec 15;13:7759. doi: 10.1038/s41467-022-35375-2 (PMC9755262; doi:10.1038/s41467-022-35375-2)
Supplement: Supplementary file 1 — Supplementary information [file 41467_2022_35375_MOESM1_ESM.pdf]

Supplementary Information for

Structural insights into p300 regulation and acetylation-dependent genome organisation

Ziad Ibrahim[1], Tao Wang[2], Olivier Destaing[2], Nicola Salvi[3], Naghmeh Hoghoughi[2], Clovis Chabert[2], Alexandra Rusu[1], Jinjun Gao[4], Leonardo Feletto[1], Nicolas Reynoird[2], Thomas Schalch[1], Yingming Zhao[4], Martin Blackledge[3], Saadi Khochbin[2] & Daniel Panne[1,*]

[1]Leicester Institute of Structural and Chemical Biology, Department of Molecular and Cell Biology, University of Leicester, Leicester, UK

[2]CNRS UMR 5309, INSERM U1209, Université Grenoble Alpes, Institute for Advanced Biosciences, Grenoble, France.

[3]Institut de Biologie Structurale, CNRS, CEA, UGA, Grenoble, France

[4]Ben May Department of Cancer Research, The University of Chicago, Chicago, IL 60637, USA.

*Correspondence: daniel.panne@leicester.ac.uk

This PDF file includes:

Supplementary Figures 1 to 7
Supplementary Tables 1 to 9

Legends for Supplementary Movies 1 and 2

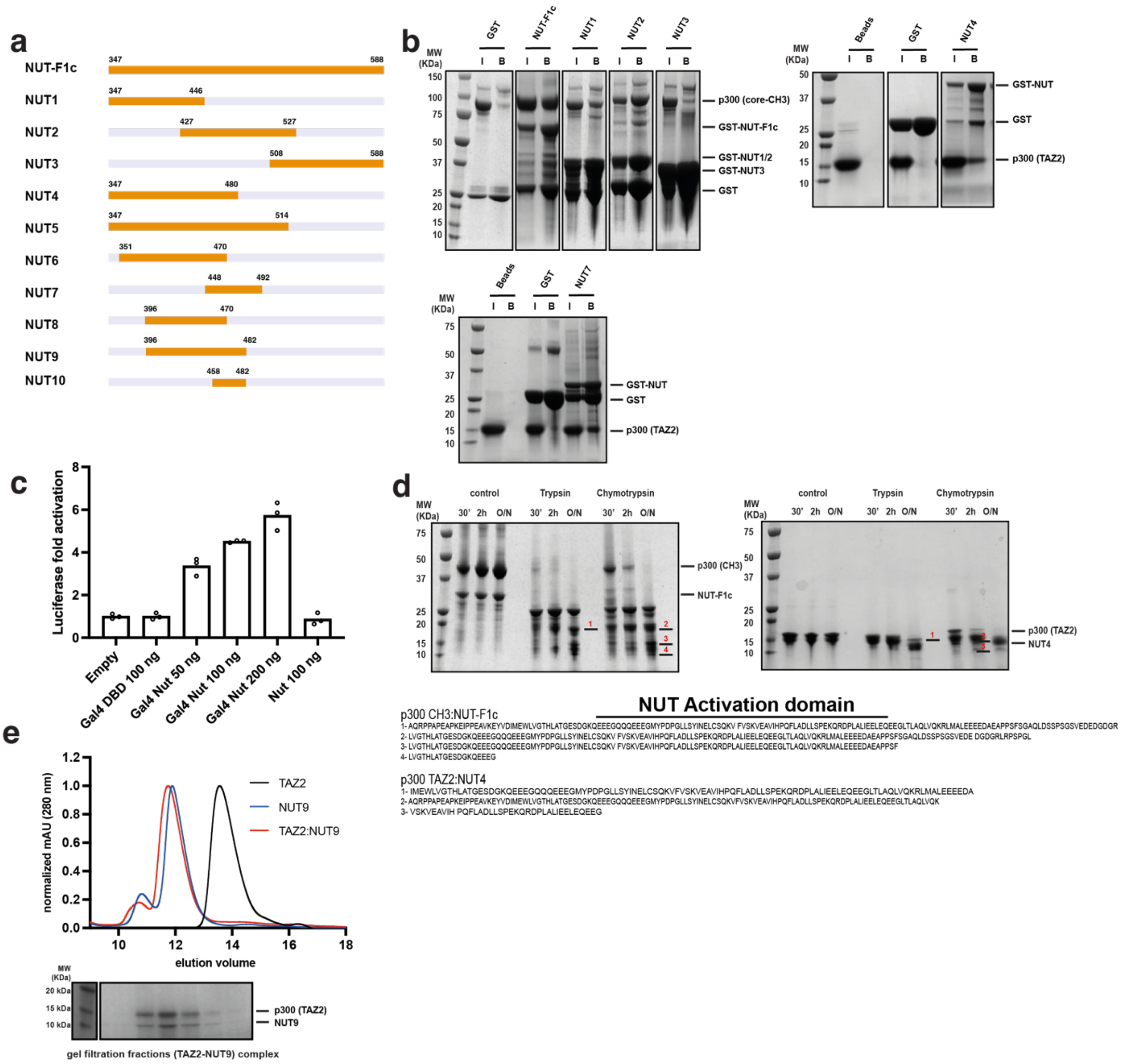

Supplementary Fig. 1. Identification of an acidic activation domain in NUT.
a, Schematic of the different NUT constructs used. Sequence coordinates correspond to NUTM1 (UNIPROT Q86Y26). **b,** GST pulldown analysis between the p300 core-CH3 or the isolated TAZ2 domain and different NUT fragments. Experiments were repeated independently three times with consistency. **c,** Luciferase reporter assay: Cos7 cells were co-transfected with a luciferase reporter plasmid and 'empty' plasmid, or containing the Gal4 DNA binding domain (DBD) or increasing amounts of Gal4-NUT-F1c plasmid or NUT-F1c lacking the Gal4 DBD. Luciferase expression was measured by luminescence (Luciferase assay kit, Stratagene). N=3 technical replicate experiments were done. Data for each replicate experiment are shown as dots (º). Bar height represents the mean signal. **d,** Limited proteolysis of the p300 CH3-NUT-F1c complex (left) or the p300 TAZ2-NUT4 complex (right). After overnight incubation with Trypsin or Chymotrypsin samples were analysed by SDS-PAGE followed by mass spectrometry. The identified NUT fragments are highlighted on the gels and their corresponding sequences are shown on the bottom. Position of the NUT AD is indicated. Experiments was done once. **e,** SEC analysis of the p300 TAZ2 domain (black), NUT9 (396-482) (blue) or the TAZ2:NUT9 complex (red). Bottom: SDS-PAGE analysis of the peak fractions of the TAZ2-NUT9 complex. The experiment was repeated two times with consistency.

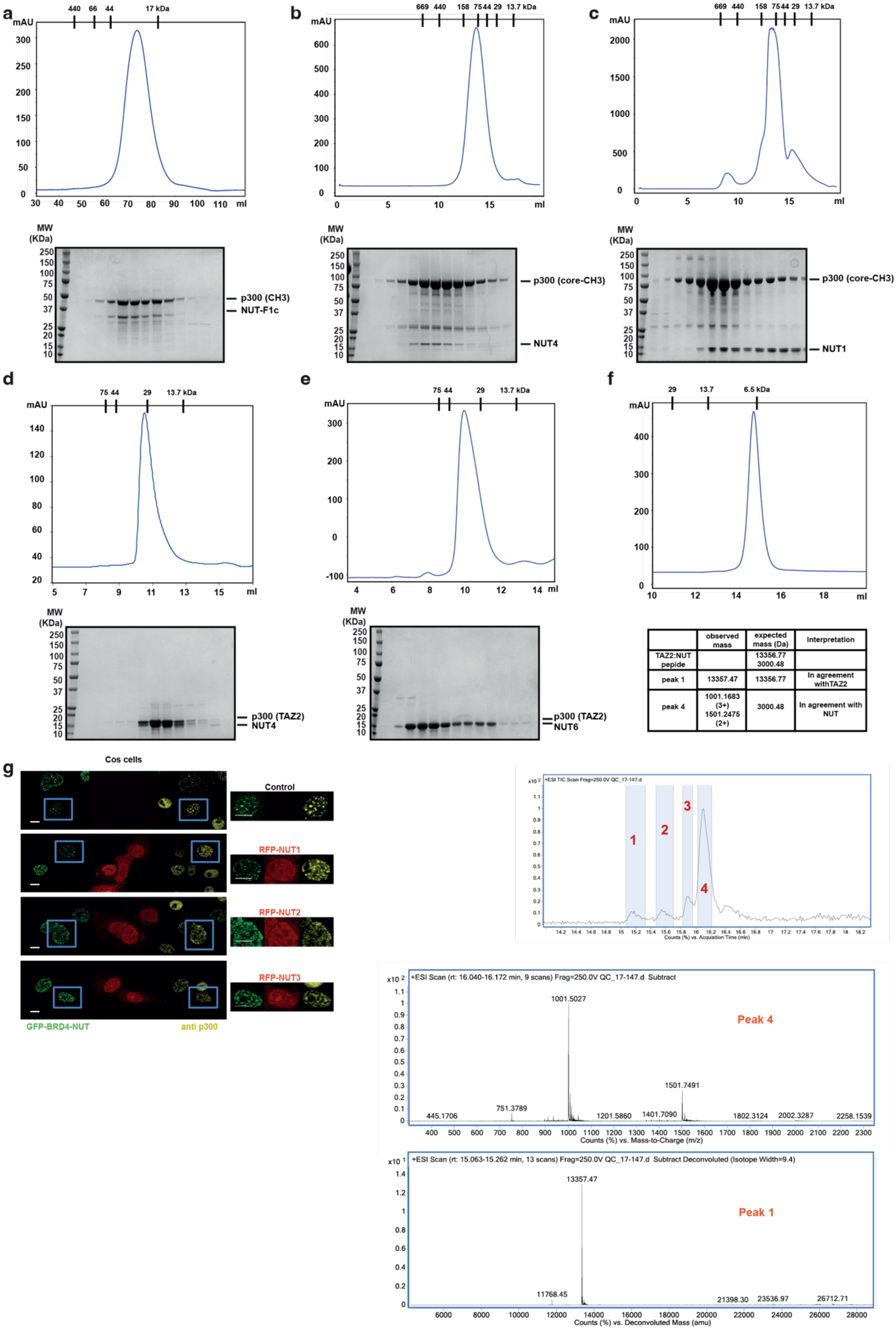

	observed mass	expected mass (Da)	Interpretation
TAZ2:NUT pepide		13356.77<br>3000.48	
peak 1	13357.47	13356.77	In agreement withTAZ2
peak 4	1001.1683 (3+)<br>1501.2475 (2+)	3000.48	In agreement with NUT

Supplementary Fig. 2. Identification of an acidic activation domain in NUT.
a, Size-exclusion chromatography (SEC) analysis of p300 CH3-NUT-F1c complex on HiLoad Superdex 200 column. **b,** SEC analysis of p300 core-CH3-NUT4 complex on Superdex 200 Increase 10/300. **c,** p300 core-CH3-NUT1 complex on Superdex 200 increase 10/300 GL. **d,** p300 TAZ2-NUT4 complex on Superdex 75 increase 10/300 GL. **e,** p300 TAZ2-NUT6 complex on Superdex 75 increase 10/300 GL. Bottom of each panel: SDS-PAGE analysis of the peak fractions. Experiments in a-e were repeated independently three times with consistency. **f,** SEC analysis of the TAZ2-NUT10 complex on Superdex 75 increase 10/300 GL column (top). Bottom: Reverse-Phase HPLC analysis of TAZ2-NUT10 peptide complex fractions followed by electrospray ionisation time of flight mass spectrometry (LC-ESI-TOF-MS). Table: Summary of the mass spectrometry analysis data showing the presence of TAZ2 (peak 1) and the NUT peptide (peak 4). **g,** Competition analysis: Cos7 cells were transfected with constructs expressing GFP-BRD4-NUT (green) alone or co-transfected with RFP-NUT1, 2 or 3 (red). Immunofluorescence with anti-p300 antibody was used to visualize condensation of endogenous p300. Experiments were repeated independently two times with consistency. Scale bars, 10 μm.

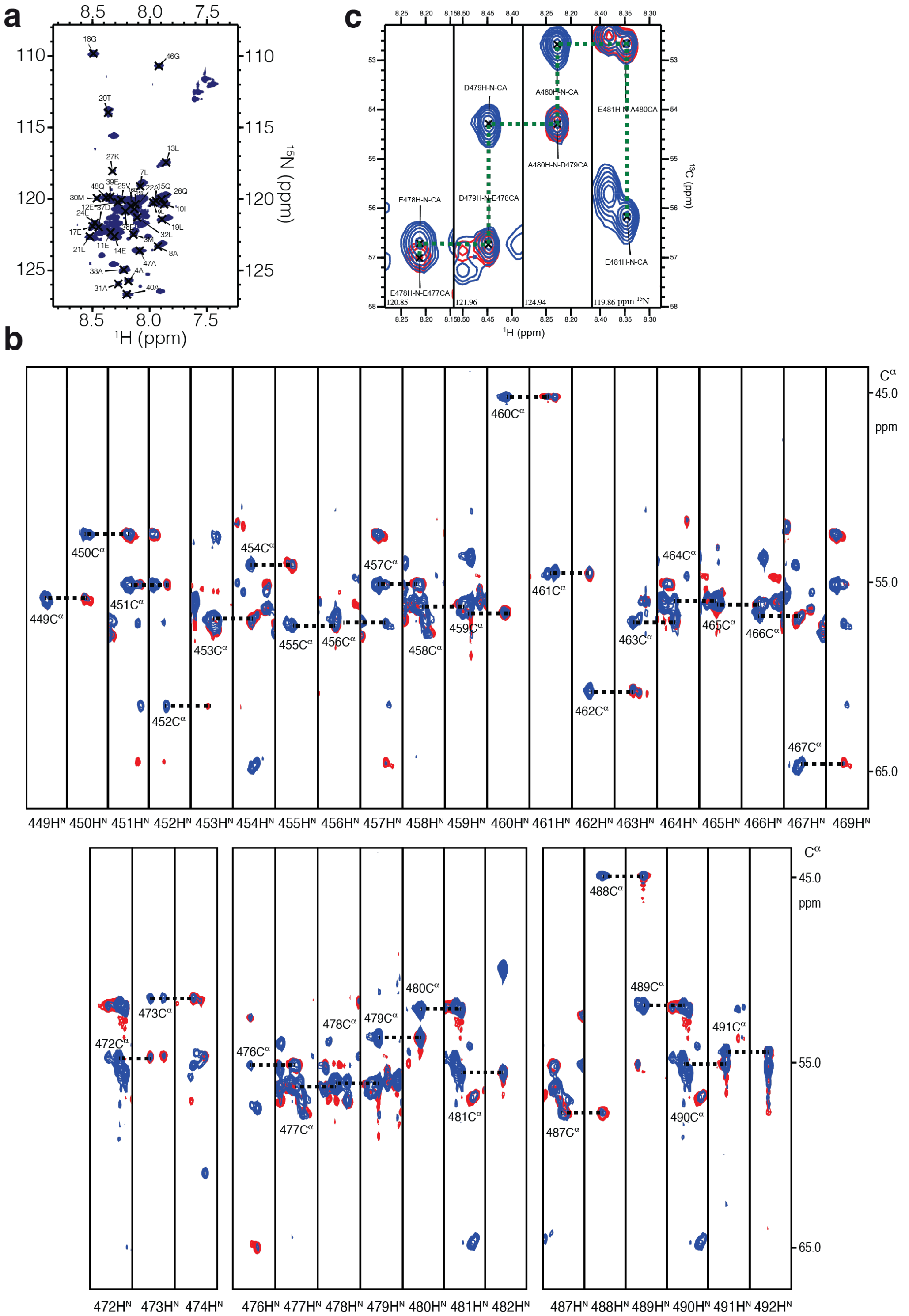

Supplementary Fig. 3. Analysis of the TAZ2-NUT interaction. a, Annotated 1H,15N HSQC spectra of NUT. **b,** Backbone assignment experiments showing magnetization transfer in HNCA (blue) and HN(CO)CA (red) experiments for the regions of NUT7(448-492) that could be assigned (experimental parameters described in Methods section). Dashed lines demonstrate correlations between neighbouring amino acids. **c,** Zoom of the same for NUT amino acids 478-481.

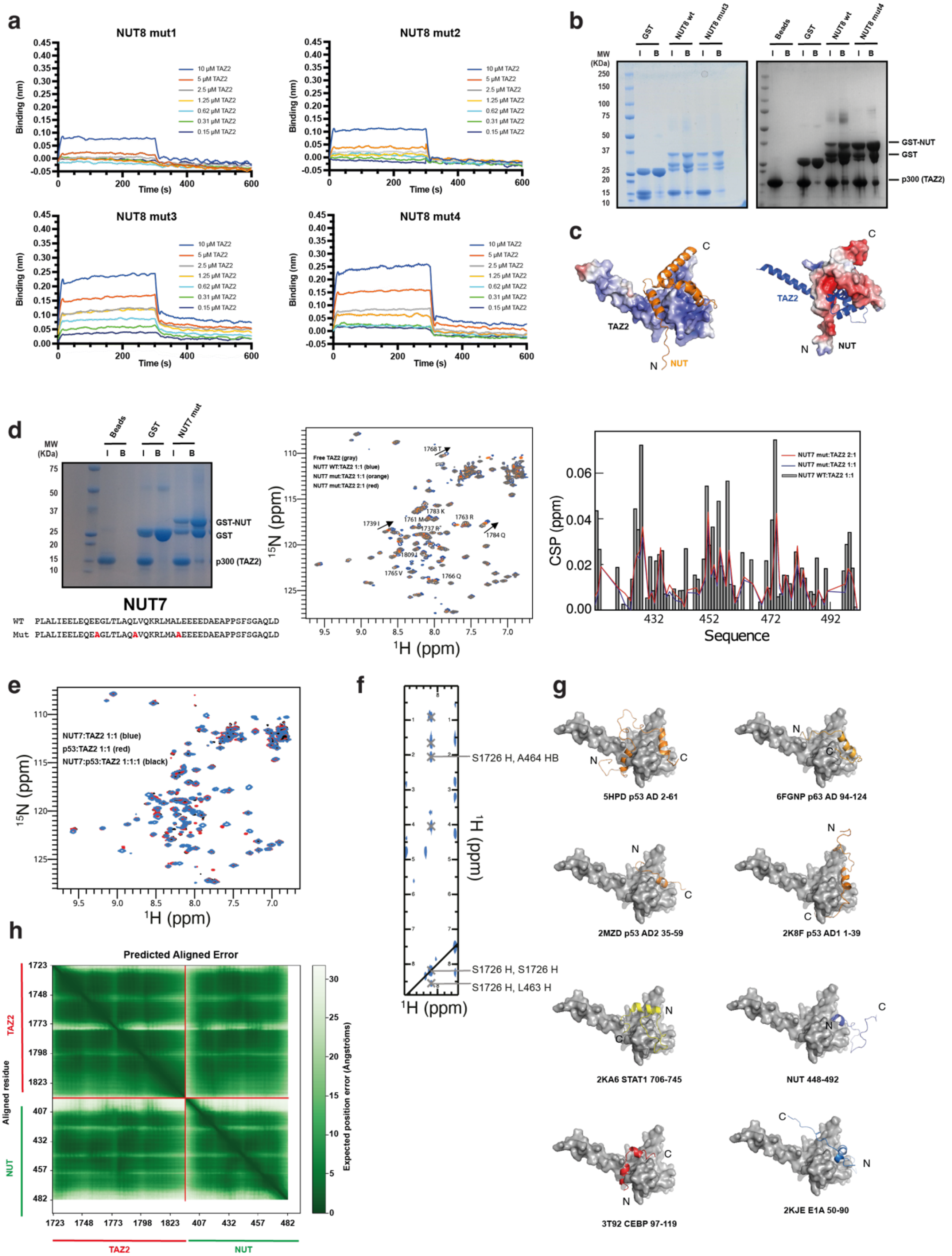

Supplementary Fig. 4. Analysis of the TAZ2-NUT interaction.

a, Representative Biolayer Interferometry (BLI) profiles of p300 TAZ2 interaction with NUT8 (396-470) mutants 1, 2, 3 and 4 from which the steady-state data shown in Fig. 3b were derived. The GST-

NUT8 construct was immobilized on the biosensors and TAZ2 was used as analyte and titrated at the indicated concentrations. **b,** GST pulldown analysis of TAZ2 and NUT8 (396-470) wild type and mutants 3 and 4. Experiments were repeated independently three times with consistency**. c,** AlphaFold model of the TAZ2-NUT complex surface rendered and coloured according to electrostatic potential. **d,** (Left) GST pulldown analysis of TAZ2 and NUT7 (448-492) mutant. Sequences of the NUT (448-492) wild type and mutant are shown below. (Middle) 1H-15N HSQC spectrum of free TAZ2 (gray) compared to a 1:1 molar ratio admixture with NUT7 (blue) or 1:1 (orange) and 2:1 (red) admixtures with the NUT7 mutant. (Right) Chemical shift perturbations (CSPs) upon adding 1 equimolar amount of NUT7 wild type (gray bars) to TAZ2 or after adding 1 (blue) and 2 (red) molar equivalents of the NUT7 mutant. Experiments were repeated independently three times with consistency. **e,** 1H-15N HSQC spectra of TAZ2 when 1 equivalent of NUT7 wild type (blue), p53 TAD (1-61) (red) and both NUT7 and p53 (black) are added. **f,** Representative ^{15}N edited NOESY spectrum using 15N,13C labelled TAZ2 and unlabelled NUT7 showing intermolecular cross peaks. **g,** Overview of different TF ADs in complex with the TAZ2 domain. The N – and C-termini of the TF ADs are indicated. **h,** Predicted Aligned Error of the TAZ2-NUT AlphaFold model. The colour at position (x,y) indicates AlphaFold's expected position error at residue x, when the predicted and true structures are aligned on residue y.

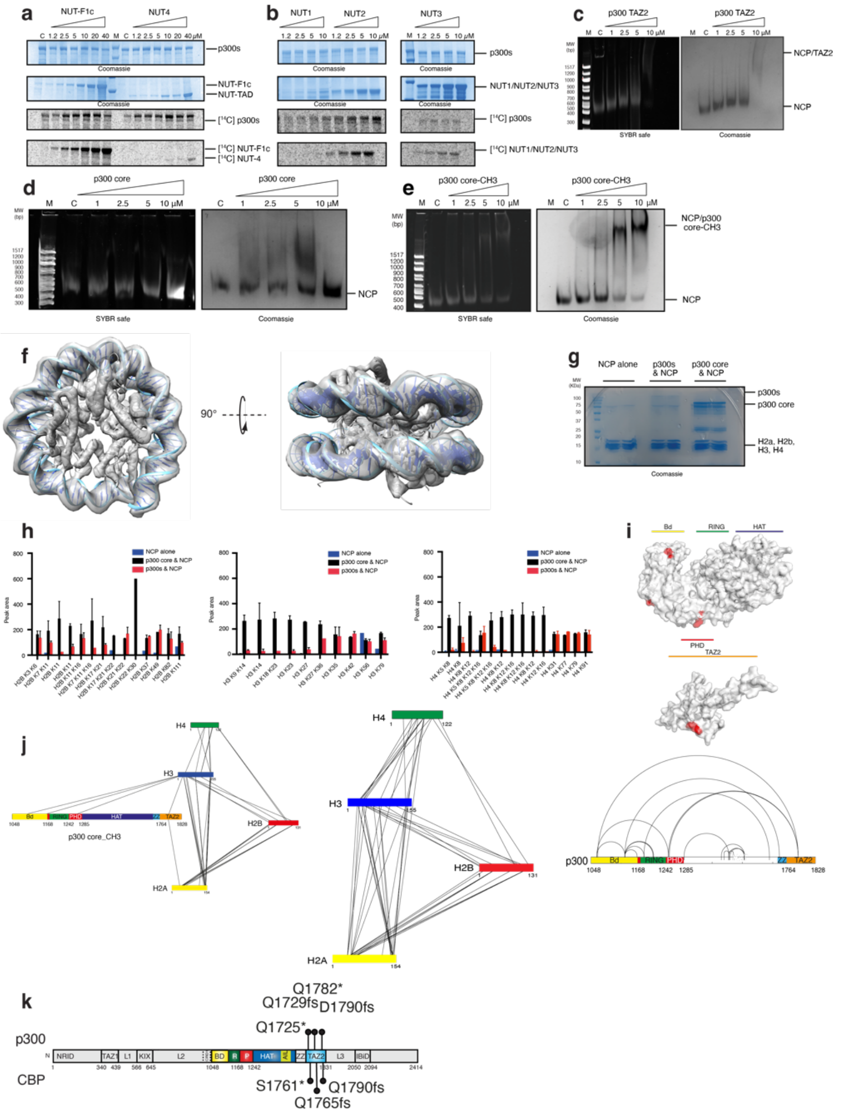

Supplementary Fig. 5. Role of the TAZ2 domain in p300 regulation and substrate acetylation. a, p300s was incubated with [^{14}C]Acetyl-CoA in the presence or absence (C: p300s alone) of (a) NUT-F1c or NUT4, **b,** NUT1, NUT2, NUT3. Samples were analysed by SDS-PAGE followed by Coomassie staining and ^{14}C phosphor imaging. **c,** EMSA analysis of NCP binding of the p300 TAZ2

domain. **d,** NCP binding of p300 core. **e,** NCP binding of p300 core-CH3. Native PAGE gels were stained with SybrSafe DNA stain or Coomassie Blue. Experiments a-d were repeated independently at least three times. Representative data are shown. **f,** CryoEM density for the NCP used in biochemical studies. An atomic model of the NCP (PDBs: 3LZ0) is docked into the density. **g,** SDS-PAGE gel showing input material used for quantitative mass spectrometry experiments. **h,** Quantitative mass spectrometry of NCP acetylation. Reconstituted NCPs were incubated with Acetyl-CoA and p300s or the p300 core. Quantification of the site-specific histone H2B, H3 and H4 acetylation by label-free HPLC/MS/MS. Data are presented as mean values +/- SD derived from n=2 independent technical replicate experiments. Source data are provided in Supplementary Data 2. **i,** Cross-link (XL-MS) analysis of the p300 core-CH3:NCP complex. p300 residues that cross-link to the NCP histones are shown in red on p300 core (4BHW) and TAZ2 (3IO2) structures. Source data are provided in Supplementary Data 1. **j,** Graphical representation of the p300:histone and histones intermolecular cross-links and intra- p300 core-CH3 cross-links. The experiment was performed once. **k,** p300 (top) and CBP (bottom) gain-of-function mutations in the TAZ2 domain.

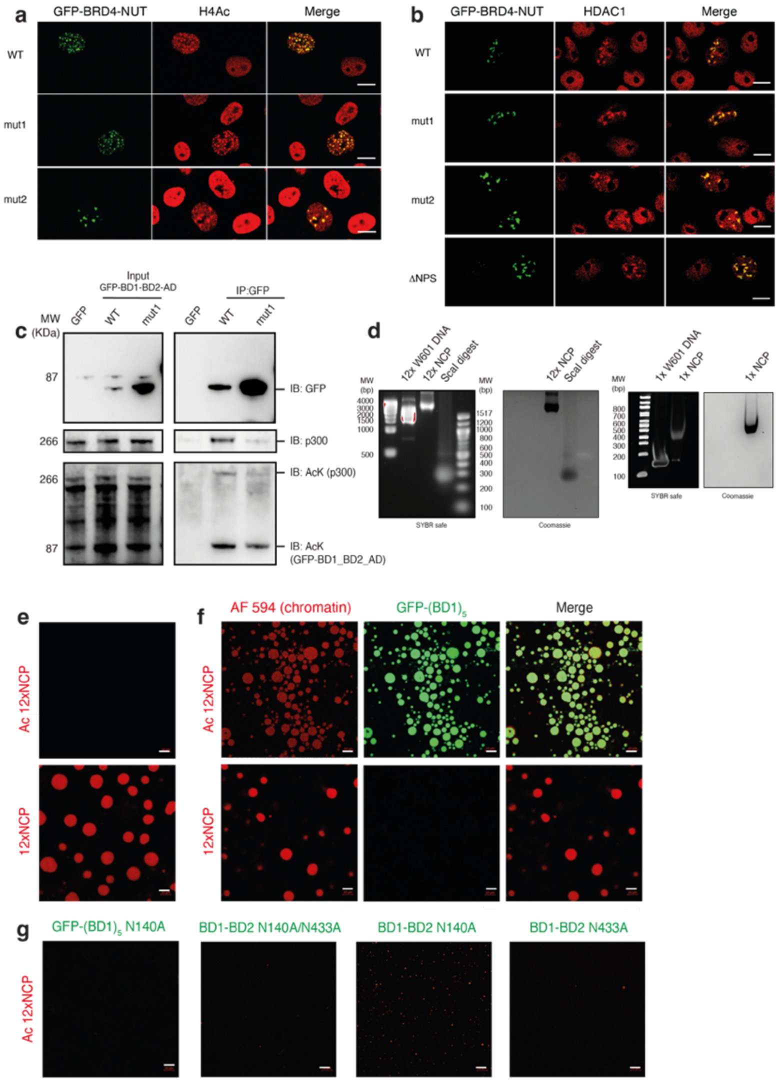

Supplementary Fig. 6. Mechanism of condensation of BRD4-NUT and p300.

a, Colocalization of BRD4-NUT condensates with acetylated H4 (H4Ac) or (**b**) with HDAC1. Cos7 cells were transfected with the indicated GFP-BRD4-NUT constructs and analysed by immunofluorescence using anti-H4Ac antibody and anti-HDAC1 antibody. Scale bars, 10 µm. **c,** Cos7 cells were co-transfected with HA-p300 and the indicated GFP-BRD4-NUT variants or GFP alone as the control. Co-immunoprecipitation (IP) experiments were performed using anti-GFP antibody and immuno-blotted (IB) for GFP, p300 and Acetylated-Lysine. (Left panel) Input samples were loaded as

a control. Experiments in a-c were independently repeated at least three times with consistency. **d,** EMSA analysis of 12x nucleosome templates assembly (left) on 1% agarose (12xNCP) native gels. The 12x nucleosome template was digested with *Sca*I restriction enzyme to isolate single NCPs. (Right) EMSA analysis of mononucleosome (1x NCP) assembly on 5% native PAGE stained with Sybr safe or Coomassie Blue. The experiment was repeated two times with consistency. **e,** Fluorescence microscopy images of AF594-labeled dodecameric nucleosome arrays. Non-acetylated 12x NCP chromatin formed droplets (red, bottom panel) that are dissolved after p300 acetylation (top panel). Scale bars, 10 µm. **f,** Acetylated and non-acetylated chromatin templates (red) in presence of GFP-labelled BRD4 $(BD1)_5$ (green). Scale bars, 10 µm. **g,** Acetylated 12x NCP chromatin with GFP-labelled BRD4 mutants. Scale bars, 10 µm. Experiment in e-g were repeated at least three times with consistency.

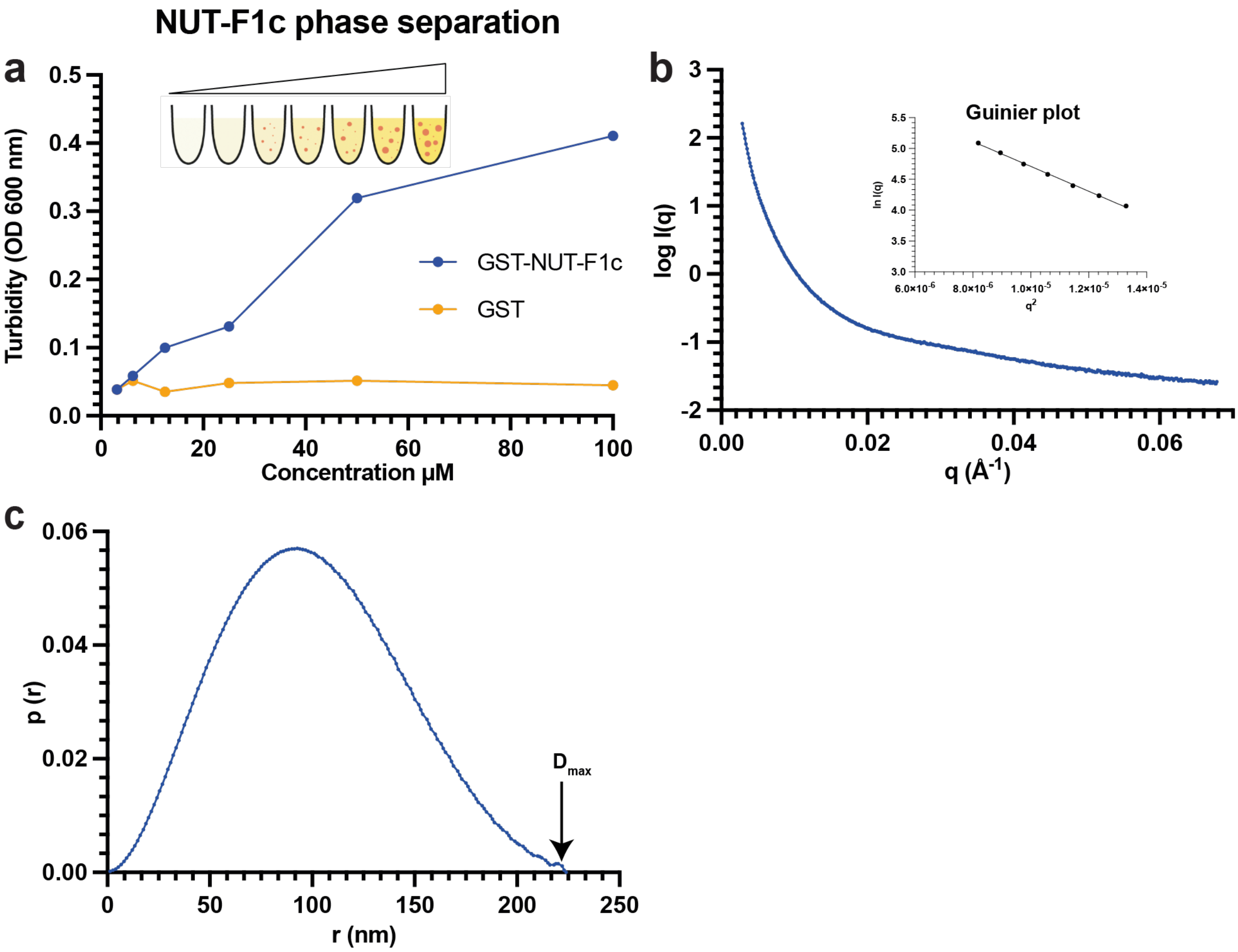

Supplementary Fig. 7. Condensation is driven by bromodomain multivalency.
a, Concentration-dependent droplet formation of GST-NUT-F1c (blue) in droplet formation buffer with 125 mM NaCl and 10% PEG-8000 measured by turbidity. **b,** Experimental SAXS profile (log intensities calculated as a function of momentum transfer) for a 100mM solution of GST-NUT-F1c in droplet formation buffer (blue). The Guinier region is inset. **c,** Distance distribution function. The experiment was repeated twice with consistency.

Supplementary Table 1 – List of NUT constructs and binding experiments

Sample	Amino acid residues	Binding*
**NUT F1c**	347-588	+
**NUT1**	347-446	+/–
**NUT2**	427-527	+
**NUT3**	508-588	–
**NUT4**	347-480	+
**NUT5**	347-514	+
**NUT6**	351-470	+
**NUT7**	448-492	+
**NUT7 mut1**	448-492	+
**NUT8**	396-470	+
**NUT8 mut1**	396-470	–
**NUT8 mut2**	396-470	–
**NUT8 mut3**	396-470	+
**NUT8 mut4**	396-470	+
**NUT9**	395-482	+
**NUT10**	468-482	+

*GST-pulldown, co-sedimentation or BLI

```
NUT7 wt:    PLALIEELEQEEGLTLAQLVQKRLMALEEEEDAEAPPSFSGAQLD
NUT7 mut1:  PLALIEELEQEAGLTLAQAVQKRLMAAEEEEDAEAPPSFSGAQLD
NUT8 wt:    GQQQEEEGMYPDPGLLSYINELCSQKVFVSKVEAVIHPQFLADLLSPEKQRDPLALIEELEQEEGLTLAQLVQKRLMALEEEEDAE
NUT8 Mut1:  GQQQEEEGMYPDPGAASAINEAASQKVAASKAEAAAHPQAAADAASPEKQRAPAGAIAAAAQAAGAALAQLVQKRLMALEEEEDAE
NUT8 Mut2:  GQQQEEEGMYPKPGALSKINELGSQKVAGSKVSAGIHGQSLADGSSPEKQRKPLGSIEKSEQKSGGSLAQLVQKRLMALEEEEDAE
NUT8 Mut3:  GQQQEEEGMYPDPGLLSYINELCSQKVFVSKVEAVIHPQFLADLLSPEKQRAPAGAIAAAAQAAGAALAQLVQKRLMALEEEEDAE
NUT8 Mut4:  GQQQEEEGMYPDPGLLSYINELCSQKVFVSKVEAVIHPQFAADAASPEKQRAPAGAIAAAAQAAGAALAQLVQKRLMALEEEEDAE
```

Supplementary Table 2 – Chemical shifts shown in figure 2a induced by adding 1 molar equivalent of TAZ2 to ^{15}N labelled NUT7(448-492)

Sequence	1H (ppm)	15N (ppm)
449Leu	8.06773	118.78799
450Ala	7.89105	123.06117
451Leu	7.94511	120.13024
455Leu	7.84131	117.27243
457Gln	7.93157	120.03990
458Glu	8.16162	120.37610
459Glu	8.46125	121.39694
460Gly	8.44341	109.65104
461Leu	7.83935	121.27930
462Thr	8.33380	113.62034
463Leu	8.58382	122.64937
465Gln	8.30271	121.70734
466Leu	8.44144	121.21074
472Met	8.46928	119.95793
473Ala	8.30164	125.96641
474Leu	8.07340	121.83051
477Glu	8.17684	120.39170
478Glu	8.19945	120.76303
479Asp	8.43618	121.91631
480Ala	8.21909	124.95127
481Glu	8.33580	119.83160
482Ala	8.19007	126.64685
488Gly	7.92783	110.73711

489Ala	8.08558	123.65890
490Gln	8.37611	119.90610
491Leu	8.31554	124.54835
492Asp	7.90591	126.48987

Supplementary Table 3 - HADDOCK docking statistics

**Distance constraints**	
Ambiguous interaction restraints (AIRs)	14
Active residues on NUT7	5
Active residues on TAZ2	9
Intermolecular NOEs	2
**Dihedral angle restraints***	
Phi/Psi	5/5
**Structural statistics**	
AIR Violations (mean ± s.d.)	2 ± 1
Ramachandran analysis	
Residues in the favoured region (%)	87%
Residues in the additional allowed regions (%)	10%
Outliers	3%
**Intermolecular energies after water refinement**	
E_{vdw} (kcal mol^{-1})	-45 ± 7
E_{elec} (kcal mol^{-1})	-303 ± 32
Buried surface area (Å^2)	1498 ± 88

*Dihedral angle restraints derived from chemical shift values using TALOS-N are applied to NUT7 only. For the TAZ2 domain, the structure in PDB 2mzd is used as input to HADDOCK.

Supplementary Table 4 - Cryo-EM data collection and map reconstruction

	**Nucleosome Core Particle**
**Data collection**	FEI Titan Krios G3
Detector	Gatan K3 direct electron detector
Voltage	300 kV
Pixel size (Å)	1.086
Nominal Magnification	81,000
Dose rate on Detector (e^- sec^{-1} px^{-1})	15
Dose rate on specimen (e^- sec^{-1} px^{-1})	17
Defocus range (µm)	-2.3 to -1.1
**Reconstruction**	
Symmetry imposed	C1
Initial particle images (no.)	80,764
Final particle images (no.)	33,202
Resolution, FSC=0.143 (Å)	6.61

Supplementary Table 5 – Quantification of condensation of BRD4-NUT variants*

GFP-BRD4-NUT	RFP-p300 positive	RFP-p300 negative
**WT**	100/100	100/100
**mut1**	99/100	89/100
**mut2**	100/100	92/100
**BD1-BD2**	16/100	0/100
**BD1**	18/100	0/100
**BD2**	99/100	99/100
**K/E**	21/100	3/100
**ΔNPS**	100/100	100/100
**ΔBID**	100/100	9/100
**BD1-BD2-AD**	99/100	1/100
**BD1-BD2-ADmut1**	0/100	0/100
**BD1-BD2-E1A**	94/100	91/100

* Foci forming cells/Total cell count

Supplementary Table 6 – Quantification of co-localization of BRD4-NUT and p300*

GFP-BRD4-NUT	Pearson's R value	Co-localization ratio
**WT**	0.81±0.01	0.90±0.02
**ADmut1**	0.84±0.01	0.87±0.02
**ADmut2**	0.76±0.02	0.90±0.01
**ΔNPS**	0.76±0.02	0.86±0.03

* At least 10 cells were analysed (n=10)

Supplementary Table 7 – Impact of Drug treatment on condensation

GFP-BRD4-NUT	JQ1	A485	TSA	CBP30	1,6-Hexandiol
**WT**	No	No	No	Yes	No
**ADmut1**	No	No	No	No	No
**ADmut2**	No	No	No	No	No
**ΔNPS**	No	No	No	Yes	No

Supplementary Table 8 – Quantification of foci formation of after CBP30 treatment*

GFP-BRD4-NUT	DMSO	CBP30
**WT**	100/100	54/100
**ADmut1**	92/100	3/100
**ADmut2**	91/100	2/100
**ΔNPS**	100/100	58/100

*Foci-forming cells/Total cell counted

Supplementary Table 9 – SAXS data analysis

Sample	GST-NUT-F1c
**Guinier Analysis**	Sample (GST-NUT-F1c)
$I(0)$ (cm^{-1})	0.8159E+03
R_g (Å)	771.84
q-range ($Å^{-1}$)	0.00286 to 0.00365
***P*(*r*) analysis**	Sample (GST-NUT-F1c)
$I(0)$ (cm^{-1})	0.8159E+03
R_g (Å)	773.00
d_{max} (Å)	2283
q-range ($Å^{-1}$)	0.0029 to 0.0678
