# Supplementary material for: Structural insights into p300 regulation and acetylation-dependent genome organisation
Source: Nat Commun. 2022 Dec 15;13:7759. doi: 10.1038/s41467-022-35375-2 (PMC9755262; doi:10.1038/s41467-022-35375-2)
Supplement: Supplementary file 3 — Description of additional Supplementary File [file 41467_2022_35375_MOESM3_ESM.pdf]

Descriptions of Additional Supplementary Files

Supplementary Movie 1: Dynamics of condensation of BD1-BD2-AD and p300. Time laps imaging. Left panel: GFPBD1-BD2-AD. Middle: RFP-p300. Right: Merge of GFP- BD1-BD2-AD and RFP-p300.

Supplementary Movie 2: Dynamics of condensation of BRD4-NUT and p300. Time laps imaging. Left panel: GFPBRD4-NUT. Middle: RFP-p300. Right: Merge of GFP-BRD4-NUT and RFPp300.

Supplementary Data 1: Cross-linking mass spectrometry data showing interactions between p300 and nucleosome core particles.

Supplementary Data 2: Mass spectrometry data showing nucleosome acetylation by p300.
