# Supplementary material for: Structural insights into p300 regulation and acetylation-dependent genome organisation
Source: Nat Commun. 2022 Dec 15;13:7759. doi: 10.1038/s41467-022-35375-2 (PMC9755262; doi:10.1038/s41467-022-35375-2)
Supplement: Supplementary file 8 — Reporting Summary [file 41467_2022_35375_MOESM8_ESM.pdf]

nature portfolio

Corresponding author(s): Daniel Panne

Last updated by author(s): 28/10/2022

Reporting Summary

Nature Portfolio wishes to improve the reproducibility of the work that we publish. This form provides structure for consistency and transparency in reporting. For further information on Nature Portfolio policies, see our Editorial Policies and the Editorial Policy Checklist.

Statistics

For all statistical analyses, confirm that the following items are present in the figure legend, table legend, main text, or Methods section.

n/a	Confirmed	
☐	☒	The exact sample size (n) for each experimental group/condition, given as a discrete number and unit of measurement
☐	☒	A statement on whether measurements were taken from distinct samples or whether the same sample was measured repeatedly
☐	☒	The statistical test(s) used AND whether they are one- or two-sided<br>*Only common tests should be described solely by name; describe more complex techniques in the Methods section.*
☒	☐	A description of all covariates tested
☒	☐	A description of any assumptions or corrections, such as tests of normality and adjustment for multiple comparisons
☐	☒	A full description of the statistical parameters including central tendency (e.g. means) or other basic estimates (e.g. regression coefficient) AND variation (e.g. standard deviation) or associated estimates of uncertainty (e.g. confidence intervals)
☐	☒	For null hypothesis testing, the test statistic (e.g. F, t, r) with confidence intervals, effect sizes, degrees of freedom and P value noted<br>*Give P values as exact values whenever suitable.*
☒	☐	For Bayesian analysis, information on the choice of priors and Markov chain Monte Carlo settings
☒	☐	For hierarchical and complex designs, identification of the appropriate level for tests and full reporting of outcomes
☐	☒	Estimates of effect sizes (e.g. Cohen's d, Pearson's r), indicating how they were calculated

Our web collection on statistics for biologists contains articles on many of the points above.

Software and code

Policy information about availability of computer code

Data collection	NMR data were collected using Topspin 3 Bruker
Data analysis	Structure validation was done with Phenix (1.19.2-4158), Structure visualisation was done with COOT 0.9, Structure renderings were done with Pymol (2.5.2_294) and UCSF Chimera 1.16, Structure analysis was done with MolProbity (4.02-528). CryoEM Movie correction was done using MotionCor2 1.4.0. CTF parameters were estimated by Gctf 1.18. Image processing was done with Relion-3.1, NMR data analysis was done with HADDOCK 2.4, MALLS data analysis was done with ASTRA software version 6.0.5.3., Biolayer Interferometry data analysis was done with ForteBio Data Analysis 9.0 software and GraphPad Prism 8.4.3, SAXS data analysis was done with Primus and GNOM softwares from the ATSAS package 3.0.5, Confocal Microscopy image analysis was done with ZEN Blue 3 acquisition and analysis software, Cross-linking mass spectrometry data analysis was done with xiNET online visualisation tool

For manuscripts utilizing custom algorithms or software that are central to the research but not yet described in published literature, software must be made available to editors and reviewers. We strongly encourage code deposition in a community repository (e.g. GitHub). See the Nature Portfolio guidelines for submitting code & software for further information.

Data

Policy information about availability of data

All manuscripts must include a data availability statement. This statement should provide the following information, where applicable:

- Accession codes, unique identifiers, or web links for publicly available datasets
- A description of any restrictions on data availability
- For clinical datasets or third party data, please ensure that the statement adheres to our policy

Chemical shifts for NUT have are available from the BMRB under accession ID 51611. Data will be released upon publication. No PDB files have been deposited. Any

other relevant data and material are available from the corresponding authors upon reasonable request.

Field-specific reporting

Please select the one below that is the best fit for your research. If you are not sure, read the appropriate sections before making your selection.

- [x] Life sciences
- [ ] Behavioural & social sciences
- [ ] Ecological, evolutionary & environmental sciences

For a reference copy of the document with all sections, see nature.com/documents/nr-reporting-summary-flat.pdf

Life sciences study design

All studies must disclose on these points even when the disclosure is negative.

Sample size	As biochemical methods are used, population sample size statistics does not apply. Wherever statistics have been derived, the number of repeat measurements and their consistency is mentioned in the figure legends. For Figures showing GST-pulldown analyses (Fig.3, Fig. 5. Fig.S1, Fig. S2, Fig. S3), appropriate controls are used to compare binding side-by-side, as is customary.
Data exclusions	No data was excluded from the analysis.
Replication	We have indicated the number of repeat measurements made and consistency of the results obtained in the figure legends. In all attempts, the repeat experiments were successful.
Randomization	Randomization is not relevant to this study, as protein samples are not required to be allocated into experimental groups. No animals or human research participants are involved in this study.
Blinding	Blinding is not relevant to this study, as protein samples are not required to be allocated into experimental groups. No animals or human research participants are involved in this study.

Reporting for specific materials, systems and methods

We require information from authors about some types of materials, experimental systems and methods used in many studies. Here, indicate whether each material, system or method listed is relevant to your study. If you are not sure if a list item applies to your research, read the appropriate section before selecting a response.

Materials & experimental systems

n/a	Involved in the study	
[ ]	[x]	Antibodies
[ ]	[x]	Eukaryotic cell lines
[x]	[ ]	Palaeontology and archaeology
[x]	[ ]	Animals and other organisms
[x]	[ ]	Human research participants
[x]	[ ]	Clinical data
[x]	[ ]	Dual use research of concern

Methods

n/a	Involved in the study	
[x]	[ ]	ChIP-seq
[x]	[ ]	Flow cytometry
[x]	[ ]	MRI-based neuroimaging

Antibodies

Antibodies used

1. anti-p300 antibody (Santa-Cruz, sc-585)
2. anti-GFP (1:5000, Covance, MMS-118R)
3. anti-H4 acetylated (1:1000, Millipore, #06-598)
4. anti-Lysine-acetylated (1:1000, Cell Signaling, #9441)
5. anti-HDAC1 (1:1000, Santa-Cruz #sc-7872)
6. anti-HA (1:4000, Abcam, ab9110)
7. Goat anti Rabbit IgG(H + L)-HRP (Bio-rad, 1:5000)
8. Goat anti-Mouse IgG (H + L)-HRP (Bio-rad, 1:10000)

Validation	anti-H4Ac: https://www.merckmillipore.com/GB/en/product/Anti-acetyl-Histone-H4-Antibody,MM_NF-06-598?ReferrerURL=https%3A%2F%2Fwww.google.com%2F<br>anti-HDAC1: https://www.scbt.com/p/hdac1-antibody-h-51?requestFrom=search<br>anti-GFP: https://www.neobits.com/covance_research_mms_118r_200_green_fluorescent_p3260766.html<br>anti-HA: https://www.scbt.com/p/ha-probe-antibody-f-7<br>anti-Kac : https://www.cellsignal.co.uk/products/primary-antibodies/acetylated-lysine-antibody/9441<br>anti-p300: https://www.scbt.com/p/p300-antibody-c-20

Eukaryotic cell lines

Policy information about cell lines

Cell line source(s)	COS7 from ATCC CR1-1651
Authentication	COS7 cell line: ATCC-CR1-1651; lot N°, 4171903
Mycoplasma contamination	COS7 cells tested negative for Mycoplasma contamination using MycoAlert Mycoplasma Detection Kit: Lonza cat N°: LT07-418
Commonly misidentified lines (See ICLAC register)	Not on the list of commonly misidentified cell lines
